# Smart Device Ownership and Use of Social Media, Wearable Trackers, and Health Apps Among Black Women With Hypertension in the United States: National Survey Study

**DOI:** 10.2196/59243

**Published:** 2024-09-09

**Authors:** Jolaade Kalinowski, Sandesh Bhusal, Sherry L Pagoto, Robert Newton Jr, Molly E Waring

**Affiliations:** 1 Department of Human Development and Family Sciences University of Connecticut Storrs, CT United States; 2 Department of Allied Health Sciences University of Connecticut Storrs, CT United States; 3 Center for mHealth and Social Media Allied Health Sciences University of Connecticut Storrs, CT United States; 4 Population and Public Health Pennington Biomedical Research Center Baton Rouge, LA United States

**Keywords:** Black women, Black, women, tracker, trackers, wearable, wearables, hypertension, hypertensive, cardiology, cardiovascular, blood pressure, social media, technology, usage, digital health, eHealth, tablet, mHealth, mobile health, app, apps, applications, survey, surveys, questionnaire, questionnaires, Health Information National Trends Survey, HINTS

## Abstract

The majority of Black women with hypertension in the United States have smartphones or tablets and use social media, and many use wearable activity trackers and health or wellness apps, digital tools that can be used to support lifestyle changes and medication adherence.

## Introduction

In the United States, Black women are disproportionately affected by hypertension, with a prevalence of 56% versus 37% among White and Hispanic women [1]. Digital health tools, including apps and wearables, can support hypertension control via lifestyle modifications and medication adherence [2]. With high social media and smartphone use in studies with local samples [3], Black women with hypertension may be poised to leverage digital tools to manage their health. However, previous studies examining digital health use among US adults with hypertension [4] have not reported use by race and sex. We examined smart device ownership and use of social media, wearable activity trackers, and health apps among Black women with hypertension in the United States.

## Methods

### Overview

We analyzed cross-sectional data from the 2022 Health Information National Trends Survey (HINTS6). Details on HINTS methodology are available on the web [5]. Briefly, civilian, noninstitutionalized adults living in the United States were sampled using a 2-stage design and completed a web-based or mail survey.

Participants reported their sex assigned at birth. Participants were asked to describe their race, and those who selected “Black or African American” (with or without other races) were included. Participants were asked if a doctor or other health professional ever told them they had high blood pressure or hypertension (yes or no). Black women reporting a diagnosis of hypertension or high blood pressure were included in the analysis.

Participants reported if they had a tablet computer or a smartphone (either vs neither), how often they visited social media sites in the past 12 months (ever vs never), and whether they used a wearable device to monitor or track health or activity in the past 12 months. Tablet/smartphone owners were asked whether they had used a health or wellness app in the past 12 months.

Participants reported their age, presence of children in their household, education, feelings about their income, and whether they had worked ≥35 hours/week in the past 30 days. Participants were asked about their confidence in finding helpful health resources on the internet (low digital health literacy: “somewhat/a little bit/not at all confident” vs adequate: “completely/very confident”). Participants reported the degree to which they have a strong sense of belonging to their ethnic, racial, or cultural group.

### Statistical Analyses

We used the survey procedures in SAS (version 9.4; SAS Institute) and replicate weights provided by HINTS6 to calculate SEs of estimates using the “delete one” jackknife replication method. Results are representative of Black women with hypertension in the US. We used logistic regression models to examine associations between participant characteristics and device ownership and mobile health use. We adjusted associations for age to account for confounding [6-8]. We assessed the assumption of linearity for age using Box-Tidwell tests.

### Ethical Considerations

HINTS6 data collection was approved by the Westat Institutional Review Board (IRB), and participants provided informed consent. The HINTS6 public-use data set is deidentified, and analyses of these data do not require additional IRB approval.

## Results

We excluded Black women with hypertension who were missing information on any variables in the analysis (n=51), resulting in an analytic sample of 409. Table 1 shows characteristics of US Black women with hypertension.

Nearly 9 in 10 (89.7%; SE 1.9%) US Black women with hypertension own a smartphone or tablet; 81.9% (SE 2.1%) used social media and 33% (SE 2.9%) used a wearable activity tracker in the past year. Of those who own smartphones or tablets, 58.7% (SE 4%) used a health or wellness app in the past year. Table 2 shows characteristics associated with smart device ownership and digital health use.

**Table 1:** Characteristics of US Black women with hypertension or high blood pressure (n=409); 2022 Health Information National Trends Survey (HINTS6).

Characteristics			Weighted % (SE)
**Age (years)**			
	18-49	32.3 (3.7)	
	50-64	40.2 (3.4)	
	≥65	27.5 (2.0)	
Works ≥35 hours per week			44.9 (3.2)
**Education status**			
	High school or less	31.8 (3.2)	
	Some college	49.8 (3.6)	
	College graduate	18.4 (2.7)	
**Feelings about present income**			
	Living comfortably on present income	26.0 (3.2)	
	Getting by on present income	40.7 (3.5)	
	Finding it very difficult/difficult on present income	33.4 (3.2)	
Has children in household			28.3 (3.9)
**Ethnic group belonging**			
	Strongly agree	55.6 (3.7)	
	Agree	20.8 (2.8)	
	Neither agree nor disagree; disagree; or strongly disagree	23.6 (3.4)	
Low digital health literacy			52.7 (3.8)

**Table 2:** Digital health activities by demographic characteristics among US Black women with hypertension or high blood pressure (n=409); 2022 Health Information National Trends Survey (HINTS6).

Characteristics		Has tablet or smartphone			Uses social media			Uses wearable activity tracker			Uses health or wellness apps^a^	
		Weighted % (SE)	Age-adjusted OR^b^ (95% CI)^c^	Weighted % (SE)		Age-adjusted OR (95% CI)^c^	Weighted % (SE)		Age-adjusted OR (95% CI)^c^	Weighted % (SE)		Age-adjusted OR (95% CI)^c^
**Age^d^ (years)**												
	18-49	96.9 (2.4)	8.5 (0.7-107.3)	98.8 (0.9)		60.1 (5.7-634.3)	38.3 (7.8)		2.0 (0.9-4.7)	66.9 (6.7)		2.6 (1.2-5.5)
	50-64	91.4 (2.9)	2.9 (1.1-7.7)	85.3 (3.1)		4.4 (2.4-7.9)	35.2 (5.7)		1.8 (0.8-4.1)	59.8 (6.2)		1.9 (0.9-4.0)
	≥65	78.9 (4.3)	(Reference)	57.1 (4.5)		(Reference)	23.4 (4.5)		(Reference)	44.2 (6.7)		(Reference)
**Works full-time**												
	Yes	96.0 (1.8)	(Reference)	90.3 (2.4)		(Reference)	46.3 (5.9)		(Reference)	66.2 (5.7)		(Reference)
	No	84.7 (2.9)	0.4 (0.1-1.0)	75.0 (3.0)		0.8 (0.4-1.7)	22.2 (3.7)		0.4 (0.2-0.8)	51.8 (5.5)		0.7 (0.3-1.6)
**Education status**												
	High school or less	77.9 (5.1)	(Reference)	66.3 (4.8)		(Reference)	20.1 (6.2)		(Reference)	34.7 (7.0)		(Reference)
	Some college	94.3 (1.7)	3.8 (1.5-9.4)	88.6 (2.3)		3.2 (1.6-6.3)	35.7 (5.1)		2.0 (0.7-5.3)	66.2 (5.2)		3.4 (1.8-6.6)
	College graduate	98.0 (0.9)	12.6 (3.9-40.7)	90.7 (2.7)		4.6 (2.2-9.5)	47.7 (6.5)		3.4 (1.2-9.7)	70.9 (6.9)		4.6 (1.6-13.3)
**Feelings about present income**												
	Living comfortably	92.4 (3.2)	(Reference)	77.3 (4.8)		(Reference)	42.1 (6.1)		(Reference)	65.8 (8.7)		(Reference)
	Getting by	87.7 (2.9)	0.5 (0.1-1.6)	80.2 (3.5)		0.9 (0.4-2.2)	28.0 (4.9)		0.5 (0.2-1.1)	55.9 (7.1)		0.6 (0.2-1.7)
	Finding it very difficult/difficult	90.1 (3.1)	0.5 (0.2-1.6)	87.5 (3.6)		1.2 (0.4-3.8)	32.1 (5.5)		0.6 (0.2-1.3)	56.4 (5.6)		0.5 (0.2-1.4)
**Children in household**												
	No	87.7 (2.2)	(Reference)	77.6 (2.6)		(Reference)	32.7 (3.2)		(Reference)	54.0 (4.8)		(Reference)
	Yes	95.0 (3.2)	1.4 (0.2-8.5)	92.7 (2.9)		1.2 (0.4-3.8)	33.8 (7.7)		0.7 (0.3-1.8)	69.2 (7.8)		1.3 (0.5-3.6)
**Ethnic group belonging**												
	Strongly agree	92.7 (2.2)	(Reference)	81.1 (3.1)		(Reference)	38.9 (4.1)		(Reference)	65.4 (5.2)		(Reference)
	Agree	80.7 (5.8)	0.3 (0.1-1.0)	79.7 (4.6)		0.8 (0.3-2.0)	33.2 (7.6)		0.7 (0.3-1.7)	52.7 (8.5)		0.5 (0.2-1.2)
	Neutral/disagree	90.7 (4.2)	0.6 (0.1-2.6)	85.5 (4.5)		1.0 (0.4-2.5)	18.8 (6.1)		0.3 (0.1-0.9)	47.0 (9.0)		0.4 (0.1-0.9)
**Digital health literacy**												
	Adequate	98.2 (1.0)	(Reference)	90.6 (2.4)		(Reference)	36.2 (4.4)		(Reference)	72.2 (5.1)		(Reference)
	Low	82.2 (3.3)	0.1 (0.02-0.5)	74.1 (3.2)		0.4 (0.2-1.0)	30.1 (4.2)		0.9 (0.5-1.6)	44.2 (4.8)		0.3 (0.2-0.7)

^a^Analysis limited to 341 participants in the analytic sample who had a tablet or smartphone and provided information on health app use.

^b^OR: odds ratio.

^c^Adjusted for age (years, continuous). Results from crude logistic regression models were largely similar. Exceptions: full-time employment and smart device ownership (crude OR 0.2, 95% CI 0.1-0.7); full-time employment and social media use (crude OR 0.3, 95% CI 0.2-0.6); children in household and social media use (crude OR 3.7, 95% CI 1.4-9.7); and ethnic group belonging (neutral/disagree) and uses health or wellness apps (crude OR 0.5, 95% CI 0.2-1.1).

^d^Models for categorical age were not adjusted for continuous age. Only 2 women aged 18-49 years did not own a tablet or smartphone and only 2 women aged 18-49 years did not use social media, limiting precision for estimates of associations between age and device ownership and social media use.

## Discussion

The majority of Black women with hypertension in the United States own smartphones or tablets and use social media, a third use wearable devices, and most mobile device owners use health apps. Younger and more highly educated women reported higher ownership and use, similar to US adults generally [6-8] and those with hypertension [4]. Ethnic belonging and low digital health literacy may also play a role.

Strengths of this study include the use of a nationally representative sample. Limitations include a lack of data on frequency of wearable tracker or health app use, degree of willingness to use digital tools for hypertension management [3], hypertension severity, and other factors that may influence device ownership and digital tool use [6-8]. Leveraging digital tools for hypertension control may be a promising strategy for the prevention of cardiovascular disease in Black women with hypertension.

## Abbreviations

HINTS: Health Information National Trends Survey
IRB: institutional review board
OR: odds ratio

## Data Availability

2022 Health Information National Trends Survey data is available for download from the National Cancer Institute [9].

## References

[1] Ostchega Y, Fryar C, Nwankwo T, Nguyen D (2020). Hypertension prevalence among adults aged 18 and over: United States, 2017-2018. NCHS Data Brief.

[2] Cao B, Gupta S, Wang J, Hightow-Weidman LB, Muessig KE, Tang W, Pan S, Pendse R, Tucker JD (2017). Social media interventions to promote HIV testing, linkage, adherence, and retention: systematic review and meta-analysis. J Med Internet Res.

[3] James DCS, Harville C (2018). Smartphone usage, social media engagement, and willingness to participate in mHealth weight management research among African American women. Health Educ Behav.

[4] Langford AT, Solid CA, Scott E, Lad M, Maayan E, Williams SK, Seixas AA (2019). Mobile phone ownership, health apps, and tablet use in US adults with a self-reported history of hypertension: cross-sectional study. JMIR Mhealth Uhealth.

[5] (2023). HINTS 6 Methodology Report. National Cancer Institute.

[6] Gottfried J (2024). Americans' social media use. Pew Research Center.

[7] Gelles-Watnick R (2024). Americans' use of mobile technology and home broadband. Pew Research Center.

[8] Vogels E (2020). About one-in-five Americans use a smart watch or fitness tracker. Pew Research Center.

[9] Download data | HINTS. National Cancer Institute.

